# The Ethnomedicine of the Haya people of Bugabo ward, Kagera Region, north western Tanzania

**DOI:** 10.1186/1746-4269-5-24

**Published:** 2009-08-31

**Authors:** Mainen J Moshi, Donald F Otieno, Pamela K Mbabazi, Anke Weisheit

**Affiliations:** 1 Department of Biological and Preclinical Studies, Institute of Traditional Medicine, MUHAS, P.O. Box 65001, Dar es Salaam, Tanzania; 2 Department of Biological Sciences, Moi University, P. O. Box 1125, Eldoret - 30100, Kenya; 3 Faculty of Development Studies, Mbarara University of Science and Technology, P.O. Box 1410, Mbarara, Uganda

## Abstract

**Background:**

The Kagera region, in north western Tanzania, is endowed with a strong culture of traditional medicine that is well supported by a rich diversity of medicinal plants. However, most of the plants in this region have not been documented nor evaluated for safety and efficacy. As an initiative in that direction, this study documented the knowledge on medicinal plant use by traditional healers of Bugabo Ward in Bukoba District.

**Methods:**

Key informants were selected with the help of local government officials and information on their knowledge and use of plants for therapeutic purposes was gathered using a semi-structured interview format.

**Results:**

In this study 94 plant species representing 84 genera and 43 families were found to be commonly used in the treatment of a variety of human ailments. The family Asteraceae had the highest number of species being used as traditional medicines. The study revealed that Malaria is treated using the highest number of different medicinal species (30), followed by skin conditions (19), maternal illnesses and sexually transmitted diseases (14), respiratory diseases (11) and yellow fever, *Herpes simplex *and peptic ulcers (10). Majority of the species are used to treat less than five different diseases/conditions each and leaves were the most commonly used part, comprising 40% of all the reports on use of plant parts. Trees comprised the most dominant growth form among all plants used for medicinal purposes in the study area.

**Conclusion:**

Bugabo Ward has a rich repository of medicinal plants and this reinforces the need for an extensive and comprehensive documentation of medicinal plants in the area and a concomitant evaluation of their biological activity as a basis for developing future medicines.

## Background

Traditional medicine is central to the provision of health care and supports well over 60% of the rural population in Tanzania [1]. Increasing population, poor economies leading to inadequate financing of the health sector and the emergence of new difficult-to-cure diseases continues to exert pressure on the health sector. Many developing countries continue to depend on traditional medicines as the main source of healthcare support for their rural populations. In the Kagera region, traditional medicine plays a significant role in the management of diseases like HIV/AIDS opportunitic infections [1]. Its place in the provision of healthcare services is well supported by the government, which enacted the Traditional and Alternative Healthcare Practice Act 2002, thus recognizing traditional medicine as being important in the healthcare of its people. However, despite legislation being in place, not much has been achieved in the documentation and evaluation of the vast resource of medicinal plants used by tradtional healers. Among over 10,000 plant species that occur in Tanzania [2,3], only slightly over 2,600 have been documented as being used in traditional medicine. Furthermore, despite these being documented there is very little that has been done in ascertaining their safety or efficacy so that they may be mainstreamed into the formal healthcare system. Given this background, there is need to increase efforts to document and evaluate the plants used in traditional medicine for safety and efficacy. The present study in Bugabo Ward, Bukoba district, north western Tanzania is therefore a continuation of on-going efforts to document medicinal plants in the Kagera region with the ultimate aim of evaluating them for biological activity and establishing how they can be mainstreamed into the social and economic development of Tanzania.

## Methods

### Description of Study area

**Figure 1:**
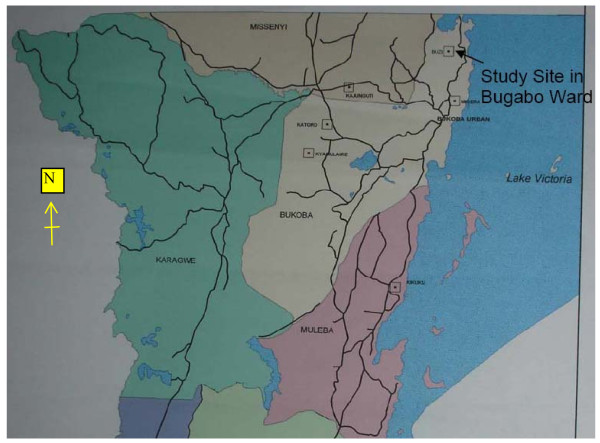
**The location of the study area in Kagera Region**.

The Kagera Region lies between 1°-2°45' S of the equator and 30°25'-32°40' East, including waters of Lake Victoria. It is made up of 7 districts: Biharamulo, Bukoba, Chato, Karagwe, Mishenyi, Muleba, and Ngara (Figure 1) and is bordered to the north by Uganda, to the west by Rwanda and Burundi and to the South by Mwanza, Shinyanga and Kigoma. It consists of a total area of 40,838 sq. km. of which 28,953 sq km. is land and 11,885 sq Km is covered by water bodies (Lakes Victoria, Ikimba and Burigi, and river Kagera and Ngono). It is Tanzania's 14^th ^largest region and occupies approximately 3.2% of the total 883,527 sq. km. land area of Tanzania mainland. Most of the region receives good rainfall and has an execellent vegetation cover, making it likely to have an abundance of medicinal plants.

### Data collection

Prior to starting the field work, one researcher travelled to Bukoba to liase with government officials to identify traditional healers who would participate in the study as key informants. The Regional Culture Office in Bukoba assisted in identifying traditional healers who were willing to participate in the study. They included a prominent traditional healer, Mr Didas Ngemera, his two brothers and parents. However, information was also recorded from other village members who volunteered to participate especially as the researchers walked through the village farms and bushes. Prior to the informants providing any information they were apprised of what the project entailed and their consent to participate in the research was then sought.

Ethnomedical information was collected in various parts of the larger Kagera region between 19^th ^February and 2^nd ^March 2008 with three days, i.e. 23^rd ^to 25^th ^being spent in Bugabo Ward. A team consisting of two cultural officers, a botanist, a pharmacologist, and a health laboratory scientist participated in the documentation of plants and collection of ethnomedical information. The information was collected using a semi-structured interview format [4] as the team walked, accompanied by the informants, through banana farms, the surrounding bushes and thickets of Buzi village and Bukombe forest, which is one of two sacred forests in Bugabo Ward that are owned by members of the Wazigu family who are traditional rulers of the area. This method was chosen because it gives one the freedom to pursue many lines of questioning [4]. Thus it was possible to complete profiles of medicinal plant species documented by collecting information on their common/local names, disease(s) treated, parts used, methods of preparation, dosage of treatment, frequency and duration of treatment and names of other plants, if any, which are mixed with each of the plants for the treatment of specific disease(s). Information was also recorded on the side effects or toxic manifestations of the herbal remedies used and whether there were antidotes that could be used against such toxic manifestations. Voucher specimens of the plants were collected and later identified by Mr. Selemani Haji of the Botany Department, University of Dar es Salaam. Duplicate vouchers are kept at the Herbaria of the Botany Department, University of Dar es Salaam and that of the Institute of Traditional Medicine, Muhimbili University of Health and Allied Sciences.

Claims made by our informants were corroborated through reports from the literature. Literature information was downloaded from the NAPRALERT Data base of the School of Pharmacy, University of Illinois at Chicago.

## Results

In the study 94 plant species representing 84 genera and 43 families were reported to be used as traditional medicines in Bugabo Ward (See additional file 1). The families Asteraceae, Rubiaceae, Fabaceae, Acanthaceae, Euphorbiaceae, Moraceae, Lamiaceae and Verbenaceae comprised 52% of all the plants documented with Asteraceae topping the list with 12 species, the highest number recorded in any one family. All the other families each had less than ten species associated with the treatment of diseases documented. In the majority of the families, only 1-3 species are used and in some cases all the species come from the same genus. The highest number of plant species recorded as being used to treat a single disease condition or problem was documented for Malaria (30), followed by skin conditions (19), maternal problems (14), sexually transmitted diseases (14), respiratory problems (11), yellow fever, *Herpes simplex *and peptic ulcers (10). Only five species were recorded as being used in the treatment of at least five different ailments each, with the remaining treating four, three, two or only one disease condition each. In many of the plants the leaves were the most commonly used part, comprising 40% of all the reports on use of plant parts. This was followed by the stem bark and aerial parts (20%) and roots (13%). The rhizome and stem sap were the least used, comprising 1% each of all the reports on use of plant parts. The dominant growth forms among the plants recorded were trees, which accounted for 30.8% (29 species), followed by herbs 28.7% (27 species), shrubs 25.5% (24 species) and climbers 14.8% (14 species).

## Discussion

Majority of the species recorded in this study are used to treat one or two disease conditions only. However, there are species e.g. *Ageratum conyzoides*, *Iboza urticifolia*, *Senecio stuhlmanii*, *Solanum nigrum*, *Trichilia emetica *and *Zehneria scabra *which are used to treat upto five different diseases each while *Alchornea cordifolia*, *Euphorbia hirta*, *Garcinia buchananii*, *Indigofera drepanocarpa*, *Pseudospondia microcarpa*, *Synsepalum ceresiferum*, *Tricalysia coriacea *and *Vernonia amygdalina *each treat four different diseases. The treatment of malaria by traditional healers in Bugabo using upto thirty different species distributed in twenty genera is quite remarkable. If febrile convulsions are also grouped as a symptom for malaria, then two more plants, *Gynura scandens *and *Lantana trifolia *can be added to the list, raising the number of plants used for treating malaria to thirty two. No other disease is treated using such a wide range of species. Malaria seems to be the most prevalent problem in this area, possibly in existence for many years and its prevalence could have led to the accumulation of such a diverse knowledge of plants with claims of antimalarial activity. Antiplasmodial activity has been reported for *Ageratum conyzoides *[5,6], *Alchornea cordifolia *[7,8], *Aspilia Mossambicensis *[9], *Clerodendron myricoides *[10,11], *Dissotis brazzae *[11], *Erythrina abyssinica *[12], *Vernonia brachycalyx *[13], *Gynura scandens *[14]and *Lantana trifolia *[15-17]. This validates the reported use of these species in the treatment of malaria by traditional healers in Bugabo.

*Clausena anisata *is known to have anticonvulsant activity [18,19] and also has angiotensin converting enzyme (ACE) inhibitory activity [20]. The former validates its reported use in the treatment of epilepsy and the latter, which is an established mechanism for lowering blood pressure, also validates its use by local healers in Bugabo in the treatment of high bood pressure. Other plants like *Canarium schweinfurthii, Dissotis brazzae*, *Isoglossa lacteal*, *Strombosia Scheffleri*, and *Whitfieldia elongata *which are used by traditional healers in Bugabo in the treatment of conditions like wounds, venereal diseases and gastrointestinal infections, had, until very recently, not been tested for biological activity. However, four of these (*Canarium schweinfurthii, Dissotis brazzae*, *Isoglossa lacteal *and *Strombosia Scheffleri*) have recently been confirmed to have antibacterial and one (*Whitfieldia elongata*) antifungal activity [21], which validates claims concerning their use as traditional medicines. In various cases documented in this study more than one plant species is used in the preparation of herbal remedies for the treatment of different ailments. For example to treat malaria, one of the herbal remedies involves mixing the roots of *Vernonia amygdalina *with the stem bark of *Sapium ellipticum *and the leaves of *Dalbergia nitidula*, *Desmodium salicifolium *and *Eriosema psoraleoides *then boiling them together and drinking the decoction. The use of more than one plant in preparing herbal preparations is normally attributed to the synergistic effect that extracts from the different plants are thought to have during treatment [22]. The prevalence in the use of leaves for the preparation of traditional herbal remedies in Bugabo Ward corresponds with what has been reported in other studies [23,24].

The dominance by trees as a source of many of the traditional medicines used in the study area can be attributed to the close proximity of Bukombe sacred forest where most of the plants used for medicinal purposes and other cultural rituals have been well conserved. One of these, *Haplocoelopsis africana *(Sapindaceae), is included in the 2006 IUCN Red List of threatened species, which makes the forest a very useful conservation area. Access into the Bukombe forest is controlled by the Wazigu family who are custodians of knowledge on rituals performed before accessing the forest. Even for this study a prayer had to be said at a shrine at the entrance of the forest before the team could be allowed to enter into the forest (see additional file 2). These rituals are well respected and together with other cultural aspects, represent a good example of how cultural traditions can contribute to the sustainable use of medicinal plant resources.

## Conclusion

From this study it is quite clear that the Haya people in Bugabo are custodians of a rich heritage of traditional medicine knowledge. This calls for more initiatives to conserve this knowledge alongside the rich repository of medicinal plants found in the study area. Also, in the light of the therapeutic claims made on many of the plants documented in this study, some of which are validated by literature reports, there is need to conduct phytochemical and biological activity studies on the plants occurring in Bugabo to generate information that could be used in future drug development.

## Competing interests

The authors bear no knowledge of competing interests in the project, and share the aspirations of the local people of Bugabo ward to bring good healthcare services to their community.

## Authors' contributions

MJM, DFO, AW, PKM, carried out the design of the study, which is being implemented in Kenya, Tanzania and Uganda. MJM interviewed traditional healers in Bukoba Rural District, compiled the information which was subsequently synthesized by MJM and DFO to this final manuscript. All authors read, revised and approved the final manuscript.

## Supplementary Material

Additional file 1 **Medicinal plants used in Bugabo Ward, Bukoba District**. A table listing plants used in Bugabo ward as traditional medicines, the methods of preparation and use. Click here for file

Additional file 2 **A photograph of one of the traditional healers, Mr. Didas Ngemera and members of his family sitting at the traditional shrine in the Bukombe sacred forest**. This photograph was taken at the shrine where a prayer was said to get permission to enter the Bukombe forest. Click here for file
