# Supplementary material for: The Ethnomedicine of the Haya people of Bugabo ward, Kagera Region, north western Tanzania
Source: J Ethnobiol Ethnomed. 2009 Aug 31;5:24. doi: 10.1186/1746-4269-5-24 (PMC2739164; doi:10.1186/1746-4269-5-24)
Supplement: Additional file 1 — Medicinal plants used in Bugabo Ward, Bukoba District. A table listing plants used in Bugabo ward as traditional medicines, the methods of preparation and use. [file 1746-4269-5-24-S1.pdf]

Table 1: Medicinal plants used in Bugabo Ward, Bukoba District

Species (Voucher No.)/Family	Vernacular name	Life form	Condition(s) treated	Part(s) used	Method of Preparation and administration
Acanthus pubescens Engl. & Turril. (MJM 3028)/**Acanthaceae**	Amatoju	S	Malaria, galactagogue in both men and cattle	R, L	The roots are cut into small pieces and boiled with water or broth/meat. Two bowls are taken per day to treat gonorrhea and syphilis
Adhatoda englerana (Lind.) C.B.Cl (MJM 3113)/ **Acanthaceae**	Ekishenda	S	Coughs treatment of warts	L, R	The leaves are put on fire to allow partial burning and then squeezed and 1 tablespoonful of the liquid obtained is given to children to treat coughs. Dry root powder is taken orally for treatment of warts
Asystasia gangetica (L.) T. Anders. (MJM 3016)/ **Acanthaceae**	Ekiingura/kinyegezi	H	Appetite booster	L	Leaves are pounded then squeezed and the juices given to patients to improve their appetite. It is also fed to calves to boost appetite and make them grow well
Isoglossa lactea Lindau (MJM 3085)/ **Acanthaceae**	Omufoka	H	Discolouration of the skin (loss of melanin), syphilis and other conditions	L	The leaves are boiled and the decoction drunk or applied topically
Thunbergia alata Sims (MJM 3030)/ **Acanthaceae**	Wankura	CL	Weakness in pregnant women	WP	The whole plant is boiled and the decoction given to pregnant women as a drink to provide energy
Whitfieldia elongata (P.Beauv.) De Wild. & T.Durand (MJM 3062)/ **Acanthaceae**	Ekigenge	S	Chicken pox, skin conditions, rectal prolapse	AP	Aerial parts are squeezed and a teaspoonful of the exudate given to a child or can also be applied topically. The exudate is also drunk for the treatment of rectal prolapse
Pseudopondias microparpa (A. Rich) Engl. (MJM 3041/3049)/ **Anacardiaceae**	Omuzilu/Mziku	T	Kidney problems, malaria, poor nutrition, peptic ulcers	SB	The fruits are used in the treatment of peptic ulcers. They are boiled together with aerial parts and roots of *Plumbago zeylanica* and aerial parts of *Oxygonum sinuatum.* A quarter to one glass of the decoction, depending on the severity of the condition is taken twice daily. A decoction made with the leaves combined with the leaves of *Ludwigia abyssinica*, *Sapium ellipticum* and *Maytenus Senegalensis* is used to treat GIT problems such as mucoid diarrhoea, peptic ulcers and chest infections

Table 1 Cont.

Steganotaenia araliacea Hochst. (MJM 3018)/**Apiaceae**	Omulalankuba	S	Herpes zoster, HIV/AIDS	AP	Dried aerial parts are powdered and applied to the skin using animal fat as a base. Decoction made from the aerial parts is also given to HIV/AIDS patients
Rauvolfia vomitoria Afzel. (MJM 3026/3114)/**Apocynaceae**	Mnyabusinde/Enyam asindi/Kinyabusinde	T	Malaria/splenomegally/abdominal colics in children/HIV/AIDS	SB, RB	For malaria the stem or root bark is boiled with water and drunk. When the decoction is given to children it makes them feel weak and hence they should also be given milk. The stem or root bark is ground and one teaspoonful of the powder is administered with porridge or tea. It can also be mixed with honey to mask the bitter taste
*Picralima nitida (*Stapf.) T.Durand & H.Durand (MJM 3075/3095)**Apocynaceae**	Mukanshe/Omukanshe	T	Maternal care during pregnancy	SB	The stem bark is pounded and soaked in water. The infusion can be used by a pregnant woman to purge spirits
Ageratum conyzoides L. (MJM 3023)/**Asteraceae**	Mwigara/Omwigara/Katabataba	H	Coughs, constipation/ Peptic ulcers and fibroids and women with difficulties to conceive	R, L	Roots are chewed fresh as an anti-acid and antiseptic. Leaves are boiled and decoction taken as tea
Aspilia mossambiscensis (Oliv.) Willd (MJM 3056)/**Compositae**	Eshurwa	H	Malaria, tonic, hypoglycaemic	WP	Aerial parts are mixed with those of *Centella asiatica*, boiled and the decoction used for the treatment of malaria
Bidens biternata (Lour.) Merr. & sherff (MJM 3155)/**Compositae**	Rwongwe/Ongwe Akakurura/Kakurura/Obukurura	H	Malaria, yellow fever	L	The leaves are boiled with water and the decoction drunk
Bidens pillosa L. (MJM 3129/3207)/**Compositae**	Akakurura/Kakurura/Obukurura	H	Wounds and warts	L	The leaves are boiled with the leaves of *Solanum nigrum* and decoction drunk until warts disappear. The leaves of the two plants can also be baked under fire and then used for wound dressing
Bidens schimperi Sch.Bip. (MJM 3039)/**Compositae**	Orwangwa	H	Yellow fever	L	The leaves are pounded, cold extracted and extractive administered orally three times a day until recovery

Table 1. Cont.

Crassocephalum mannii (Hookf) Milne (MJM 3020/3060) /**Compositae**	Akagango-Akake	H	Febrile convulsions in children, malaria or heart problems	AP	Aerial parts are pounded and boiled in water and the decoction administered orally. It is also applied topically e.g. by bathing in it.
Crassocephalum vitellinum (Benth.) S. Moore (MJM 3046) /**Compositae**	Ekishenda	CLH	Peptic ulcers, prevent miscarriage	AP	The aerial parts are boiled and the decoction is taken regularly as a treatment for peptic ulcers. A decoction made with aerial parts mixed with the roots of *Alchornea cordiflia*, *Sapium ellipticum*, *Acanthus pubescens* and *Emilia javanica* is used to prevent miscarriage
Gynura scandens O.Hoffm. (MJM 3083) /**Compositae**	Ekizimya muliro	CLH	Fever, febrile convulsions	L	The leaves are squeezed or pounded and then boiled with water. The decoction is given to children to drink and at the same time the juice extracted from the leaves is applied on the body using a sponge to lower high temperatures
Senecio stuhmannii Klatt (MJM 3043/3134) /**Compositae**	Eirarire/kikarabwe/kikarabo/Omugango	CL H	Wounds, swellings, coughs, stiff neck, poisoning	L	The leaves are pounded and used as an antiseptic dressing for wounds or rubbed on the neck. For coughs the leaves are baked with salt and chewed. The leaves and charcoal made from the stem are used as an antidote for poisoning
Vernonia amygdalina Del. (MJM 3101) /**Compositae**	Omubilizi	S	Febrile convulsions, fever, malaria and mastitis in cows	L, R	Leaves are squeezed and the exudate administered. For the treatment of mastitis in cattle, the leaves are pounded and put in drinking water. For the treatment of malaria and febrile convulsions a decoction of the roots is mixed with the stem bark of *Sapium ellipticum* ,and the leaves of *Dalbergia nitidula*, *Desmodium salicifolium* and *Eriosema psoraleoides* and drunk
Vernonia brachycalyx O.Hoffm. (MJM 3047/3161) /**Compositae**	Mkuraijwa/Omuwa	S	Guinea worm, backache	AP	Aerial parts are burnt and the ash applied to the knee where the worm is haboured. For treatment of backache – the leaves are mixed with those of *Pappea capensis*, boiled and about 2 tablespoonfuls of the decoction given to the patient three time a day

Table 1 Cont.

Markhamia zanzibarica (DC.) Engl. (MJM 3089)/**Bignoniaceae**	Omushambya	T	Yellow fever, dysentery, mucoid diarrhea	SB	The stem bark is mixed with that of *Mangifera indica* , *Maesopsis eminii* and *Erythrina abysinica* then boiled and a half a glass of the decoction administered daily for the treatment of yellow fever, or mixed with *Bidens pilosa* , *Psidium guajava* , *Harungana madagascariensis*, and the rusty yellow part of banana leaves and then boiled and decoction taken
Canarium schweinfurthii Engl. (MJM 3036)/**Burseraceae**	Muubani wa kiume/omubafu	T	Malaria, syphilis	SB	The bark is ground into powder and boiled and decoction drunk
Garcinia buchananii Bak. (3065)/**Clusiaceae**	Msharazi	S	Venereal diseases, dysentery, HIV/AIDS, malaria	SB, R	The stem bark or roots are boiled with a small amount of water and concentrated to half volume or mixed with roots of *Tragia furialis* for the treatment of malaria. The decoction of the roots or stem bark is also used as treatment for venereal diseases
Bryophyllum pinnatum (Lam.) Kurz. . (MJM 3107)/**Crassulaceae**	Kikugwa/chikugw a	H	Cold/flu and coughs in both children and adults	L	The succulent leaves are wrapped in banana leaves and baked under fire. The baked leaves are then squeezed and the exuding juice administered to patients orally
Momordica foetida Schumach. (MJM 3090)/**Cucurbitaceae**	Orwirwa/orwikur a	CL	Skin conditions/ Chicken pox	AP	The aerial parts combined with those of *Whitfieldia elongate* are crushed together and the juice squeezed out and administered to children with chicken pox. The exudates are also applied topically to treat skin lesions resulting from chicken pox
Zehneria scabra (L.f.) Sond. (MJM 3025/3150) /**Cucurbitaceae**	Akabindizi	CL.	Skin diseases, gonorrhea, syphilis, cleansing uterus before a child is delivered, malaria	L	Leaves are boiled and decoction drunk. To treat malaria a decoction is made from boiling the leaves combined with the roots of *Trichilia emetica*, *Alchornea cordifolia* and *Sapium ellipticum*, of which one cup is administered three times a day for up to 7 days
Fimbristylis hispidula (Vahl) Kunth. (MJM	Ndeju	H	Fractures	L	The leaves are pounded and soaked in water and as much as possible of the infusion taken

3045)/**Cyperaceae**					
Scleria bulbifera A. Rich. (MJM 3127) /**Cyperaceae**	Enki	H	Urinary obstruction (prostate?)	RZ	Underground rhizomes pounded, squeezed and the exudate drunk

Table 1. Cont.

Alchornea cordifolia (Schumach.) Müll. Arg. (MJM 3109)/ **Euphorbiaceae**	Mjururuzi/omujururuzi	T	Gonorrhea, syphilis, febrile convulsions and malaria	R, L	Leaves are mixed with those of *Sapium ellipticum*, *Kigelia africana*, *Zehneria scabra*, *Acanthus pubescens*, *Emilia javanica* and *Oxygonum sinuatum*, boiled and the decoction taken daily for the treatment of malaria
Euphorbia hirta L. (MJM 3022/3184) / **Euphorbiaceae**	Omushesha/Kahyaburimbe	H	Galactagogue, hypertension, warts, cataract (local application)	AP	The milky exudates are applied topically to the eyes to remove cataracts. The aerial parts are mixed with those of *Solanum nigrum*, *Bidens pilosa*, and *Oxygonum sinuatum* and boiled. The decoction is then orally administered daily for the treatment of warts. A decoction of aerial parts alone is used by mothers to enhance lactation
Euphorbia tirucali L. (MJM 3029) / **Euphorbiaceae**	Ikonora	S	Skin disease and galactagogue	AP	The aerial parts are pounded or boiled with water then applied topically (for abscess) or taken orally as a galactagogue.
Macaranga monandra (L.) Muell. (MJM 3082) / **Euphorbiaceae**	Mshakoenyonyi/ Mshakwanyonyi	T	Low haemoglobin level	SB	The stem bark is boiled to make a decoction in combination with the leaves of *Bidens pilosa* and green coffee beans
Sapium ellipticum (Krauss) Pax (MJM 3130/3105) / **Euphorbiaceae**	Orushasha	T	Malaria	L, SB, R	A decoction is made by boiling the roots with those of *Trichilia emetica* and *Alchornea cordifolia* together with the leaves of *Zehneria scabra*. The decoction is then administered orally three times a day for up to 7 days
Tragia furialis Bojer (MJM 3014) / **Euphorbiaceae**	Engenyi (Upupu)	CL	Impotence, boost libido	L	The leaves are mixed with the roots of *Elaeodendron buchananii* , *Vernonia brachycalyx* and *Desmodium ramosissimum* and boiled in water. One glass of the decoction is then administered daily until effect can be observed
Abrus precatorius L. (MJM 3013)/ **Fabaceae**	Kaligaligo	CL	Coughs	L	Leaves are pounded together with ginger to make a thick paste which is squeezed and the exuding liquid given to children for coughs
Cassia mimosoides L. (MJM 3031/3060) /**Fabaceae**	Bushenganziru/Akanwe akake	H	Fracture/antibacterial, cleaning of the	AP	Aerial parts are pounded and mixed with animal fat and applied topically or can be taken orally. They are

			uterus by pregnant women		also pounded with the leaves of *Cassia polytricha* (Fabaceae) and the paste tied around a fracture to promote healing

Table 1 Cont.

Desmodium ramosissimum G.Don. (MJM 3053) **/Fabaceae**	Kasikasiki	H	Low libido	R, L	Roots and leaves are boiled with those of *Tragia furialis* and *Clerodendrum buchanannii* in water and one glass of the decoction administered daily to increase libido
Entada abyssinica A. Rich. (MJM 3102) **/Fabaceae**	Mwiganjula	T	Swollen stomach (Ascites)	SB	Stem bark boiled with water and decoction taken orally
Erythrina abysinica DC (MJM 3245) **/Fabaceae**	Omulinzi	T	Alcoholism, malaria	FL, SB	The flowers are mixed with those of *Dracaena fragrans* dried and then ground into a powder which is then put into the brew or alcoholic drink being taken by the patient without his/her knowledge. The stem bark is boiled with water and the decoction used for malaria
Indigofera drepanocarpa Taubert (MJM 3055) **/Fabaceae**	Mbabazi	CLH	Chronic malaria, venereal diseases, wounds, tonic	R	The roots are boiled in water and a cup of the decoction taken three times a day
Sesbania macrantha Phil. & Hutch. (MJM 3106) **/Fabaceae**	Omubimba	S	Syphilis and miscarriages in women		The leaves are boiled and one cup of the decoction taken three times a day. The leaves can also be mixed with those of *Zehneria scabra*, and the roots of *Sapium ellipticum* and *Alchornea cordifolia*, boiled and the decoction taken orally
Dovyalis abyssinica (A. Rich.) Warb. (MJM 3033)/ **Flacourtiaceae**	Omuyebe	T	Cough	SB	The bark is boiled in water together with the leaves of *Abrus precatorius* and the decoction administered orally
Leonotis nepetifolia (L.) Ait.f. (MJM 3104)/**Lamiaceae**	Kitaterante	H	Malaria, febrile convulsions	AP	The aerial parts are boiled in water and the decoction taken orally three times daily for up to 5 days
Ocimum gratissimum L. (MJM 3123) **/Lamiaceae**	Kashwagara	H	Blotting, wounds	L	One teaspoonful of dry leaf powder is taken with tea or food
Tetradenia riparia (Hochst..) Codd (MJM 3112/3125) **/Lamiaceae**	Mkono wa nkhanda/kiswija/ Omushunshu	H	Ulcers, tonsils, wounds, malaria, insect bites	L	A decoction is made from the leaves and drunk or the leaves are pounded and used for dressing wounds. For malaria the leaves are boiled with water and a glass of the decoction taken three times a day

Hugonia castaneifolia Engl. (MJM 3074)/**Linaceae**	Kinyamarobo	CLW	Given to pregnant women to improve health of fetus	AP	The aerial parts are cut into small pieces and boiled when fresh with water. The decoction is administered to pregnant women regularly

Table 1 Cont.

Anthocleista grandiflora Gilg (MJM 3241)**/Loganiaceae**	Mgabaigana	T	Malaria	SB	The dried stem bark is boiled and a cup of the decoction taken three times a day for 5-7 days depending on severity
Hibiscus cannabinus L. (MJM) 3054)/**Malvaceae**	Kakungiri/Lukingiri	H	Wounds, cleansing uterus	AP	The leaves are pounded and used for wound dressing. They are also boiled with water and the decoction is taken orally presumably to treat intra-uterine infections
Hibiscus fuscus Garcke (MJM 3017) /**Malvaceae**	Omusinga	S	Polio	L	The leaves are pounded then mixed with fat (Ghee) and massaged on the affected area
Dissotis brazzae Congn. (MJM 3098)/**Melastomataceae**	Katuntunu	H	Syphilis, malaria	AP	The aerial parts are mixed with those of *Solanum nigrum*, boiled and the decoction administered orally
Dissotis rotundifolia (Sm.) Triana (MJM 3038/3244) /**Melastomataceae**	Obweee/Obweheee/Marwa	H	Giddiness, malaria and alcoholism	AP, L	The leaves are boiled with water and the decoction administered orally or the leaves are squeezed and the juice expressed is taken with beer/alcoholic drinks to help control alcohol dependency
Trichilia emetica Vahl. (MJM 3066)/**Meliaceae**	Omushunguti/omujunguti	T	Epilepsy, malaria, TB, gonnorhea, syphilis	SB, L, R	Stem bark/leaves are boiled in water and decoction administered. In the treatment of gonnorhea and syphilis the roots are boiled together with the roots of *Alchonea cordifolia, Sapium ellipticum, Zenheria scabra, Acanthus pubescens, Emilia javanica* and *Oxygonum sinuatum* and the decoction drunk.
Bersama abyssinica var *engleriana* (Gürke) F. White (MJM 3108)/**Melianthaceae**	Muhinguza/Omuhinguza	T	Body spasms, parasitic worm infestation	R, SB	A decoction is made by boiling the roots and stem bark
Xymalos monospora (Harv.) Baill. (MJM 3040)/**Monimiaceae**	Kamagaliko	T	Coughs	L	Leaves are pounded together with ginger to make a thick paste or squeezed and the juice produced given to children for coughs
Antiaris toxicaria (Pers.) Lesch. (MJM 3063)/**Moraceae**	Omujuju	T	Used for superstitious purposes	WP	The whole plant is used as a charm
Ficus asperifolia Miq. (MJM	Ekijuhuju/Omuku	S	Throat ulcers	L	Leaves are boiled and the decoction drunk

3071) /**Moraceae**	ruguta				
Ficus exasperata Vahl (MJM 3091/3131) /**Moraceae**	Msomolo	T	Warts, throat conditions	SP	The stem sap is applied locally to treat warts while the leaves are boiled and decoction taken for throat conditions

Table 1 Cont.

Ficus thonningii Bl. (MJM 3086) /**Moraceae**	Mugwa/Mugwi	T	Swollen painful feet (athlete foot rot)	L	The leaves are pounded, soaked in water and the infusion used for washing feet
Maesa lanceolata Forssk. (MJM 3080)/**Myrsinaceae**	Mzilanyama/Omuzilanyama	T	Small pox and chicken pox	L	The leaves of the plant are pounded and the extract applied topically as an antiseptic for the treatment of rashes.
Eugenia capensis (Eckl. & Zeyh.) Sond. (MJM 3094/3066)/ **Myrtaceae**	Omtukuza	S	Removal of excessive water from the uterus of pregnant women (hydrofoetalis)	AP	The leaves are boiled with leaves from plants like *Vernonia bradycalyx* and *Strombosia scheffleri* and the decoction is drunk by pregnant women to reduce excessive amniotic fluid
Ochna Schweinfurthiana F.Hoffm (MJM 3076) /**Ochnaceae**	Mkomanshanje	T	Chest problems and skin rashes	L	Extracts from the leaves are applied topically for skin rashes while for chest problems the leaves are boiled with water and the decoction taken orally
Strombosia scheffleri Engl. (MJM 3032)/**Olaceae**	Mmarara/omumarara	T	Treatment of diarrhea	SB	Stem bark is boiled with water and the decoction taken orally
Jasminum dichotomum Vahl (MJM 3084)/**Oleaceae**	Kigoyagoye	CLW	Severe malaria	L	The leaves are boiled and the decoction drunk or they are dried and ground into a powder which is then licked
Ludwigia abyssinica A. Rich. (MJM 3024)/**Onagraceae**	Runyamiga/Omulalankuba	H	HIV/AIDS, fungal infections	L	The leaves are boiled and the decoction drunk
Plumbago zeylanica L. (MJM 3015)/**Plumbaginaceae**	Enkira/Enkila	S	Peptic ulcers	AP	The aerial parts are boiled in water with the stem bark of *Pseudonpondias microcarpa* and aerial parts of *Oxygonum sinuatum* and the decoction drunk
Oxygonum sinuatum (Meisn.) Dammer (MJM 3061) /**Polygonaceae**	Nyawaija/Enyawaija	H	Wounds, warts	L	The leaves are burnt and the ash licked
Portulaca spp. (MJM 3099)/ **Portulacaceae**	Lumwaga	H	Blotted stomach. Used by pregnant women to reduce	AP	The aerial parts are boiled in water and used as tea. The leaves have an almost sweet or slightly salty taste.

			gas from the stomach		
Maesopsis eminii Engl. (MJM 3034)/**Rhamnaceae**	Omuumura	T	Constipation	SB	The bark is soaked in a local brew and the infusion taken orally
Rubus rigidus Sm (MJM 3103) **Rosaceae**	Mkelele	S	Syphilis	AP	The aerial parts are boiled in water and the decoction drunk

Table 1 Cont.

Chassalia umbraticola Vatke (MJM 3027)/**Rubiaceae**	Mwataibale/mwatabazimu/kivumbasi	S	Mental illness	L	The leaves are mixed with the leaves of *Euphorbia tirucali* and *Ocimum suave* then boiled and the decoction given to patient in the morning to drink
Hymenodictyon parvifolium Oliv. subsp *parvifolium* (MJM 3100) /**Rubiaceae**	Mazi ge nkeremeka (infant stool)	CLW	Malaria, chest problems involving difficulty to breath in young children	AP	The aerial parts are pounded and boiled then given to children to drink
Oxyanthus speciosus DC. (MJM 3070) /**Rubiaceae**	Mwanikibira	T	Low heamoglobin levels, tonic, dizziness	L	Leaves are boiled and the decoction is drunk
Pavetta refractifolia K.Schum. (MJM 3079) /**Rubiaceae**	Mwanga	S	Mental confusion	AP	A decoction is made from the roots combined with those of *Vernonia brachycalyx* and drunk
Rytigynia uhligii (K.Schum. & K.Krause) Verd. (MJM 3044) /**Rubiaceae**	Rulokola	S	Paralysed limb	L	Leaves are pounded and then applied or massaged on affected area
Tricalysia coriacea (Benth.) Hiern. (MJM 3064/3170) /**Rubiaceae**	Mshekela kibira/ Mwanikibira/Omumwanikibira	T	Yellow fever (jaundice); Skin diseases, epixstasis, malaria	L, R	Leaves/roots are boiled and decoction drunk
Vangueria apiculata K.Schum. (MJM 3048) /**Rubiaceae**	Kirongomani/Mtigunda	S	Diabetes	SB, L	A decoction is made using the leaves mixed with those of *Dracaena steudneri*
Vangueria infausta Burch. (MJM 3139) /**Rubiaceae**	Amabungo/mabungo/ Kirongomani/Mtigunda	T	Malaria, Diabetes	L, SB	The fruits and seeds are burnt and then eaten or the leaves boiled in water together with leaves or stem bark of *Dracaena steudner*i (Agavaceae) and the decoction drunk
Teclea nobilis Del. (MJM 3049)/**Rutaceae**	Omuzo	S	Worms	L	The leaves are pounded and dried into a powder and mixed with the leaf powder of *Sapium ellipticum.* The resultant powder is then licked

Clausena anisata (Willd.) Benth. (MJM 3046) /**Rutaceae**	Kata tera munyanya	CLH	Epilepsy, High blood pressure	R	The roots are boiled in water and the decoction administered three times daily
Allophylus chaunostachys Gilg (MJM 3081)/**Sapindaceae**	Orutete/Akatete	CLW	Colds, swelling of legs and warding off spirits	L	The leaves are boiled and the decoction drunk as a cold remedy and to treat swollen legs. Bathing with an infusion of the leaves is believed to ward off bad spirits
Haplocoelopsis africana F. O.Davis (MJM 3078)/**Sapindaceae**	Mkuzanyana	T	Malaria and spasms	SB	Dried stem bark is boiled with water and the decoction taken three times a day

Table 1 Cont.

Synsepalum brevipes (Bak.) Pennington (MJM 3035)/ **Sapotaceae**	Omukaraito/mkaraito	T	Malaria	SB	The stem bark is ground and boiled and the decoction administered twice daily for one week
Synsepalum ceresiferum (Welw.) Pennington (MJM 3077/3097) / **Sapotaceae**	Omumo	T	Malaria, venereal diseases, TB and urinary tract infections	SB	The stem bark is boiled in water and decoction taken orally
Capsicum frutescens L. (MJM 3170)/**Solanaceae**	Karumali	H	Insomnia, impotence	L, FR	The leaves are boiled and the decoction used to treat insomnia while a decoction of boiled fruits is used as treatment for impotence
Solanum nigrum L. (MJM 3021)/ **Solanaceae**	Shwiga/Eshwiga/ Ntura/Butura	H	Worms e.g. hookworms (safura), ring worms; warts, blood pressure, bed wetting in children	L, FR	For ringworm the leaves are pounded and applied topically. The leaves can also be pounded and baked under fire before being used for dressing a wart. To treat wetting of the bed in children, ripe fruits are given to them to eat
Pteryogota mildbraeii Engl. (MJM 3067)/**Sterculiaceae**	Muzimya/Muzimia	T	For spiritual functions		The leaves are boiled with water and used for bathing as a way to ward off bad spirits
Trema orientalis (L.) Blume (MJM 3052)/**Ulmaceae**	Omuwuwe/ Omuuwe	S	Yellow fever, low levels of haemoglobin	AP	Leaves are pounded and boiled with the leaves of *Combretum collinum* (Combretaceae) and *Erythrina abbyssinica* . (Fabaceae), and the decoction used to treat yellow fever. An infusion from the leaf is used as a haematinic
Centella asiatica (L) Urb. (MJM 3059)/**Umbelliferae**	Mbatama/Kutikwimo	H	Malaria and recurrent fever, headache	AP, L	Aerial parts mixed with a local brew and patient given a bowl or cup to drink to treat malaria and recurrent fever. For headaches, the leaves are rubbed on the forehead

Giradinia diversifolia (Link) Friis (MJM 3019)/**Urticaceae**	Akajumbura akaki/Akanyango	S	Breathing problems	L	The leaves are pounded, squeezed and the juice administered orally
Clerodendrum cephalanthus Oliv. Var. cephalanthum/**Verbenaceae**	Maiko	S	Eye problems	R	The roots are boiled in water with the roots of *Bridelia micrantha* and the ensuing steam directed into the eyes through a narrow aperture
Clerodendrum myricoides (Hochst.) Vatke. (MJM 3198) /**Verbenaceae**	Omurajio	S	Malaria, febrile convulsions, abdominal colics	SB, R	The stem bark or roots are boiled in water and half a teaspoonful of the decoction is administered orally
Lantana trifolia L. (MJM 3042) /**Verbenaceae**	Kashekelauku	S	Febrile convulsions	L	The leaves are pounded, boiled with water and the decoction administered to children

CL = Climber, CLW = Climber wood, CLH = Climber herb, H = Herb, S = Shrub, T = Tree, AP Aerial part, FL = Flower, L = Leaves, R = Root, RB = Root bark, RZ = Rhizome, SB = Stem bark, SP = Stem sap, WP = Whole plant, FR = Fruit
