# Supplementary figures and images for: The Ethnomedicine of the Haya people of Bugabo ward, Kagera Region, north western Tanzania
Source: J Ethnobiol Ethnomed. 2009 Aug 31;5:24. doi: 10.1186/1746-4269-5-24 (PMC2739164; doi:10.1186/1746-4269-5-24)

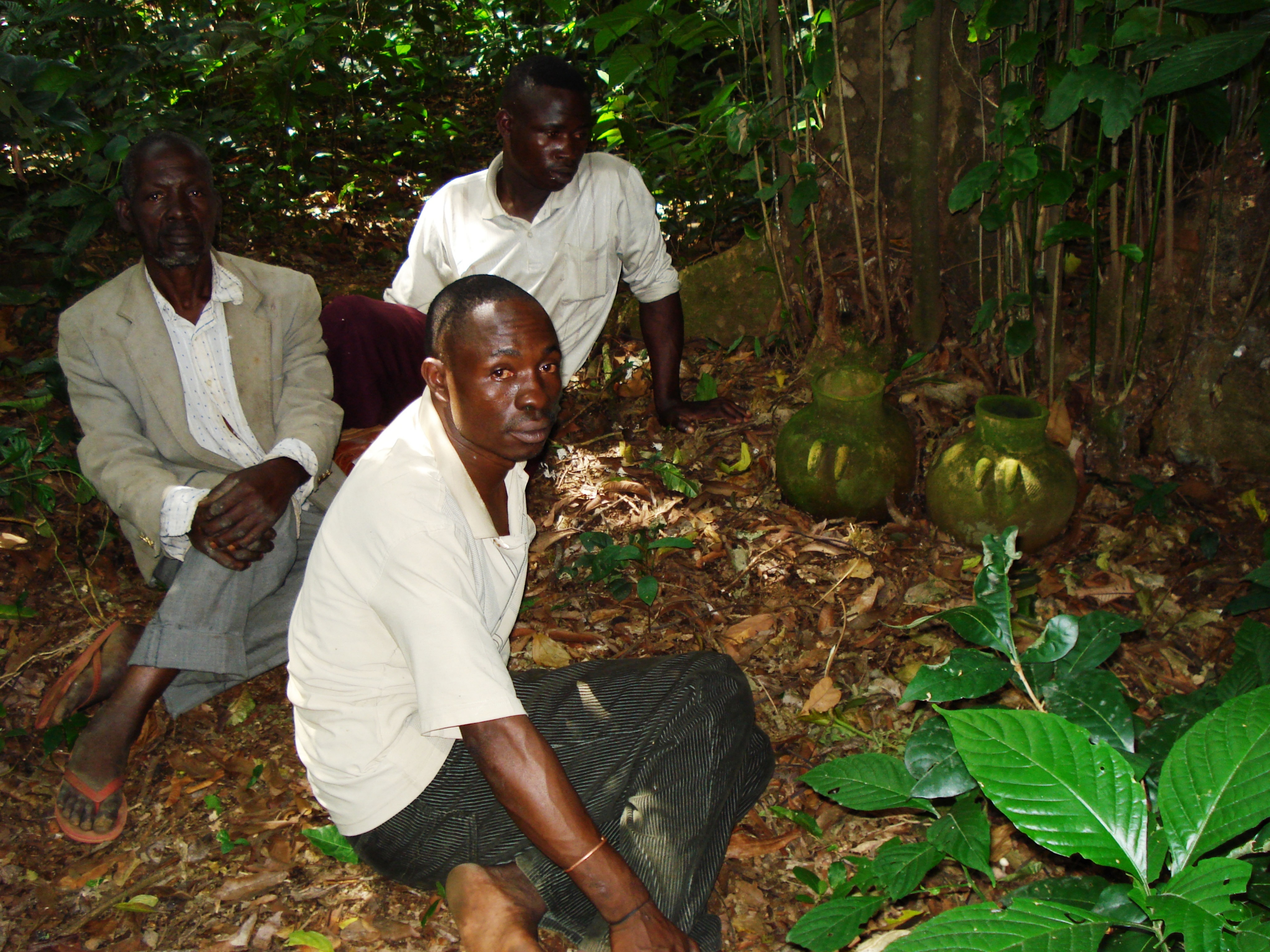

Supplement: Additional file 2 — A photograph of one of the traditional healers, Mr. Didas Ngemera and members of his family sitting at the traditional shrine in the Bukombe sacred forest. This photograph was taken at the shrine where a prayer was said to get permission to enter the Bukombe forest. [file 1746-4269-5-24-S2.pdf]
